# Quantitative analysis of two isoflavones in *Pueraria lobata *flowers from eleven Chinese provinces using high performance liquid chromatography

**DOI:** 10.1186/1749-8546-5-14

**Published:** 2010-04-23

**Authors:** Meicun Yao, Yiting Liao, George Q Li, Francis CP Law, Yi Tang

**Affiliations:** 1 School of Pharmaceutical Sciences, Sun Yat-sen University, Guangzhou 510006, Guangdong, PR China; 2 Herbal Medicines Research and Education Centre, Faculty of Pharmacy, University of Sydney, NSW 2006, Australia; 3 Department of Biological Sciences, Simon Fraser University, Burnaby, BC V5A 1S6, Canada

## Abstract

**Background:**

*Pueraria lobata *flower (*Gehua*) is a medicinal herb to treat intoxication, hepatic and gastrointestinal tract lesion induced by alcohol. This study aims to develop a new HPLC method for the determination of two major isoflavones in *P. lobata *flowers, namely tectoridin and 6"-O-xylosyl-tectoridin.

**Methods:**

A high performance liquid chromatography (HPLC) method with a C_18 _column (250 mm × 4.6 mm, 5 μm) was developed for the quantitative analysis of tectoridin and 6"-O-xylosyl-tectoridin, the main isoflavone components in *P. lobata *flower. A simple gradient of acetonitrile/water (0 min 15:85; 35 min 50:50; 36 min 15:85; 40 min 15:85; v/v) was used, and 265 nm was selected as detection wavelength. Tectoridin and 6"-O-xylosyl-tectoridin were used as the external standards in quality control of *P. lobata *flower for the first time. The method was applied to practical use in quality assessment of eleven batches of *P. lobata *flower samples in Chinese herbal medicine market.

**Results:**

The peak area response was linear for tectoridin in the 11.8-236.4 μg/mL range with a correlation coefficient of 0.9996 (P < 0.001), and for 6"-O-xylosyl-tectoridin in the 10.33-185.99 μg/mL range with a correlation coefficient of 0.9984 (P < 0.001) respectively. The average recoveries were 102.7-103.7% for tectoridin and 95.7-103.2% for 6"-O-xylosyl-tectoridin (RSDs < 3%), and the intra-day and inter-day RSDs of the two components were less than 2%. This HPLC method was applied to assess the quality of *P. lobata *flower from eleven provinces in China. *P. lobata *flowers from northern China contained 26.46-43.28 mg/g of tectoridin and 30.90-48.23 mg/g of 6"-O-xylosyl-tectoridin comparing to 10.00-19.81 mg/g of tectoridin and 11.08-37.03 mg/g of 6"-O-xylosyl-tectoridin in those from southern China.

**Conclusion:**

The results showed that *P. lobata *flowers from northern China contained more tectoridin and 6"-O-xylosyl-tectoridin than those from southern China.

## Background

*Pueraria lobata *(Willd) Ohwi is a common Chinese medicinal plant that belongs to the *Leguminosae *family. While the *P. lobata *root (*Gegen*) is beneficial for cardiovascular diseases, the *P. lobata *flower (*Gehua*) is used to treat intoxication, hepatic and gastrointestinal (GI) tract lesion induced by alcohol [1]. *P. lobata *flower reduces ethanol absorption by the GI tract [2,3] and modulates the immune and endocrine systems to alleviate the damage caused by alcohol to the hepatic and GI functions. *P. lobata *flower has anti-diabetic [4], anti-stress [5], anti-viral [6] and antioxidant [7] properties and induces apoptosis in human neuroblastoma cells [8]. It is also used to treat rectal ulcer and bleeding [9].

**Figure 1:**
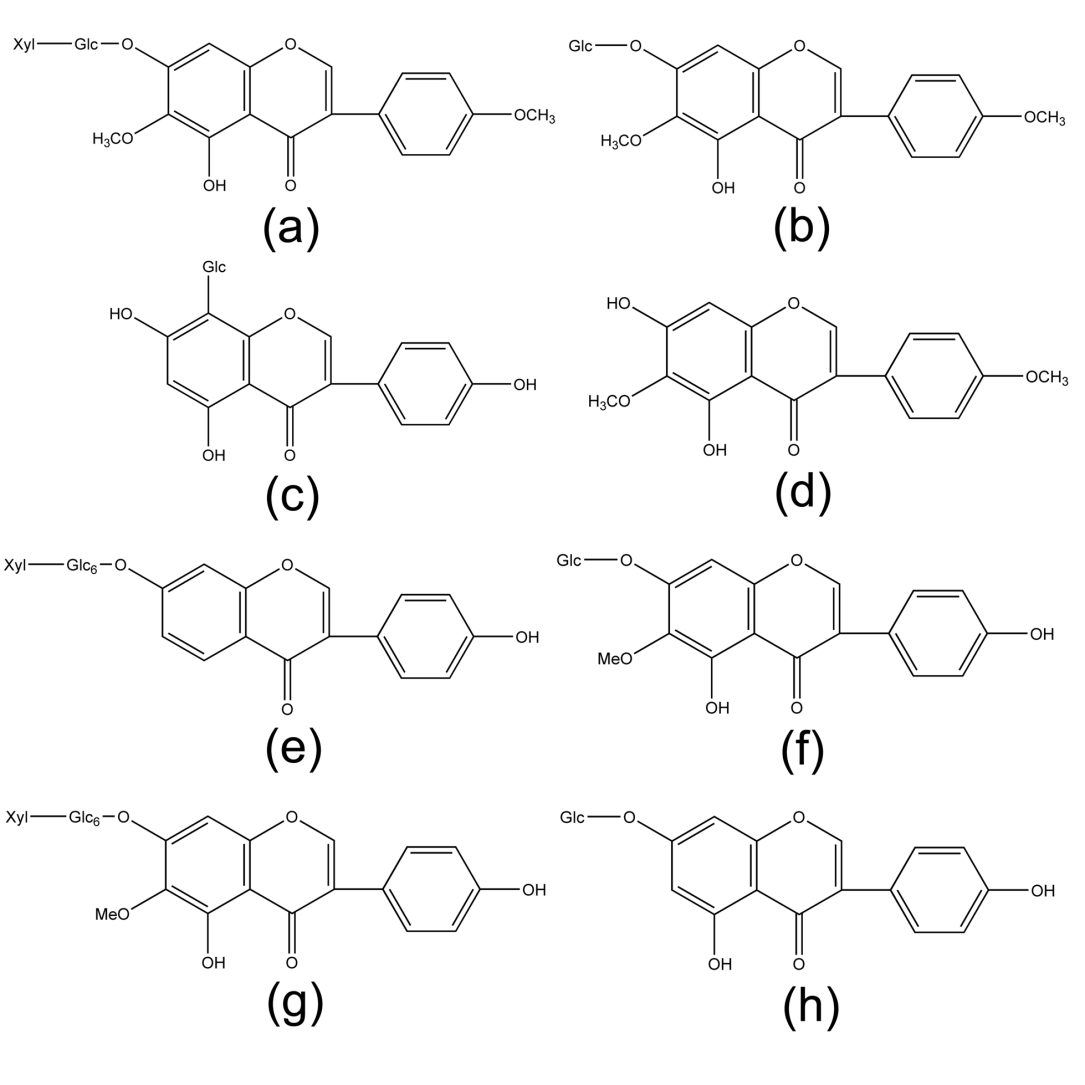
**Chemical structures of isoflavones in *P. lobata *flower**. (a) kakkalide, (b) kakkalidone, (c) puerarin, (d) irisolidone, (e) 6"-O-xylosyl-glycitin, (f) tectoridin, (g) 6"-O-xylosyl-tectoridin, (h) genistin.

Most of pharmacological effects of *P. lobata *flower have been attributed to its isoflavone components [2,3,5,6,8-10], e.g. kakkalide, kakkalidone, puerarin, irisolidone, 6"-O-xylosyl-glycitin, tectoridin and 6"-O-xylosyl-tectoridin (Figure 1); thus, determination of isoflavone is crucial for the quality control of *P. lobata *flower [11].

Chemical components in *P. lobata *flowers undergo changes under storage conditions. Tectoridin is the major isoflavone component during the first-5-year storage and nearly no kakkalide is detected [12]. In this study, we also found that tectoridin was the major component of ethanol (70%) extract of *P. lobata *flower. Tectoridin is used as a marker compound for the quality evaluation of *Belamcanda chinensis *in the Chinese Pharmacopoeia [13]. While tectoridin is not a characteristic component of *P. lobata *flower, it may be used as the marker compound for *P. lobata *flowers. Tectorigenin, the major metabolite of tectoridin, showed potent pharmacological effects on ethanol-induced diseases [2,5,8].

Several methods with isoflavone determination for *P. lobata *flower have been reported [12,14-16]. For example, an ultraviolet (UV) spectrophotometry method to determine the isoflavone content with kakkalide as the marker was developed [16]. However, puerarin is often used instead of kakkalide in China where kakkalide is not widely available. The UV method is not satisfactory as the maximum absorbance wavelength of puerarin (250 nm) is different from that of kakkalide (270 nm). A high performance liquid chromatography (HPLC) analysis with chloroform, methanol and distilled water was used to determine the freshness of *P. lobata *flower [12]. Another HPLC method with isoflavones genistin and genistein as external standards was developed for quality control of the three isoflavones in *P. lobata *flower, namely 6"-O-xylosyltectoridin, tectoridin and tectorigenin [15].

A new HPLC analytical method was developed in this study, which was with a reversed phase column and a UV detector for the quantitative determination of the two major isoflavones in *P. lobata *flower, namely tectoridin and 6"-O-xylosyl-tectoridin. The same kinds of isoflavone standards were used as the external standards, whereby two major isoflavones from eleven batches of *P. lobata *flowers were determined.

## Methods

### Materials and chemicals

*P. lobata *flowers from eleven provinces in China were purchased from Guangzhou Medicinal Materials Co. (Guangzhou, China). The samples were from eleven locations of production, namely Hebei, Shanxi, Shandong, Henan, Jiangsu, Anhui and Hubei (provinces in northern China) as well as Jiangxi, Hunan, Guangxi and Guangdong (provinces in southern China). The authenticity of production were certified by Jun Wang, Associate Professor, School of Pharmaceutical Sciences, Sun Yat-sen University (Guangzhou, China), by observation of the shapes and microscopic characteristics, and properties tests according to Guangdong Chinese Materia Medica Standards [17]. Voucher specimens were deposited in the School of Pharmaceutical Sciences, Sun Yat-sen University (Guangzhou, China).

Reference standards of tectoridin and 6"-O-xylosyl-tectoridin were purchased from the National Institute for the Control of Pharmaceutical and Biological Products of China (Beijing, China). The purity for all reference standards was over 99%. HPLC grade acetonitrile, methanol and analytical grade ethanol for isoflavones extraction were obtained from Hanbang Chemical Reagent Co. (China). HPLC-grade water was obtained from Millipore Elix 3 Progard 2 Water System (Millipore, USA).

### Preparation of standard solutions

Stock solution of tectoridin (236.4 μg/mL) was prepared by dissolving 11.82 mg of tectoridin in 50 mL of 80% (v/v) methanol solution. Stock solution of 6"-O-xylosyl-tectoridin (186.0 μg/mL) was prepared by dissolving 9.3 mg of 6"-O-xylosyl-tectoridin in 50 mL of 80% (v/v) methanol solution. The stock solutions were stored at 4°C and used within one month. Working solutions were prepared by serial dilutions of the stock solution; 47.3, 94.6, 189.1 μg/mL of tectoridin and 37.2, 148.8, 186.0 μg/mL of 6"-O-xylosyl-tectoridin were used as the quality control solutions. The standard curve was plotted according to the linear least-squares using the peak areas (Excel 2000, Microsoft, USA).

### Sample preparation

Dry samples were pulverized to fine powder, 8 g of which was accurately weighed and immersed in 80 mL of 70% (v/v) ethanol solution for 60 minutes. It was then refluxed in a water bath (88°C) for 60 minutes and filtered; the residue was further refluxed twice in 64 mL of 70% (v/v) ethanol solution for 60 minutes under the same condition. Combined filtrates were evaporated to dryness and further vacuum dried in an oven. About 20 mg of the powder was dissolved in 50 mL of 80% (v/v) methanol with sonication. Once cooled to room temperature, the sample solution was filtered through a 0.45 μm syringe membrane filter (Millipore, USA). The filtrate (10 μL) was injected into Waters 717-1525-2996 HPLC system (Waters, USA) for analysis.

### Statistical analysis

Linearity analysis was performed according to linear least-squares regression with Excel 2000 (Microsoft, USA). Calculation of relative standard deviation (RSD) was also carried out with Excel 2000 (Microsoft, USA). One-way ANOVA (*α *= 0.05) was applied in the means comparison of extraction yields and isoflavone determination results of samples from northern and southern provinces with SPSS 16.0 (IBM, USA). P < 0.05 was considered statistically significant.

### Instruments and the chromatographic conditions

The Waters HPLC system was equipped with a binary pump, auto-sampler and Waters 2487 dual wavelengths absorbance detector (Waters, USA). All separations were performed on a GRACE C_18 _column (250 mm × 4.6 mm, 5 μm) (W.R. Grace, USA). Mobile phase was a gradient of acetonitrile/water (0 min 15:85; 35 min 50:50; 36 min 15:85; 40 min 15:85; v/v). Flow rate was 0.8 mL/min. Quantification was performed with a UV detector (Waters, USA) at 265 nm. Injection volume was 10 μL. There was a 10-minute interval between sample injections. The column was kept at ambient temperature.

### Calibration

Obtained with seven concentrations in triplicates, the calibration curve was plotted according to the linear regression analysis of the integrated peak areas (*y-axis*) versus concentrations (*x-axis*, μg/ml) of tectoridin and 6"-O-xylosyl-tectoridin. Limit of detection (LOD) and limit of quantification (LOQ) were determined with standard solution at a signal-to-noise ratio of three and ten respectively.

### Precision

We studied the intra-day and inter-day variability to evaluate the precision of the method. Three solutions (high, medium and low concentrations) containing 30.5, 60.9, 118.5 μg/mL of tectoridin and 38.1, 69.4, 129.7 μg/mL of 6"-O-xylosyl-tectoridin were prepared. The quantity of each component was determined by the respective calibration curve. RSD was used to measure precision. The inter-day reproducibility test was carried out on three different days.

### Repeatability

Six independently prepared extracts of *P. lobata *flower from Henan province, China were analyzed and the repeatability was assessed by calculating the RSDs in six measurements.

### Recovery

Recovery studies were carried out by spiking three concentrations of mixed standards of tectoridin and 6"-O-xylosyl-tectoridin to the *P. lobata *flower sample from Henan. The spiked samples were extracted and analyzed. Recovery (%) = (A_s_-A)/A_a _*100%. A_s _refers to the amount of tectoridin or 6"-O-xylosyl-tectoridin found after spiking of the mixed standards whereas A refers to the amount of those found before spiking. And A_a _refers to the amount of reference standards actually added to the sample. The average recoveries of every spiking concentration and the RSDs of recoveries in each concentration groups were calculated.

## Results

### Optimization of the chromatographic conditions

Ultraviolet scanning from wavelength 200 nm to 400 nm was carried out for *P. lobata *flower 70% ethanol extract and tectoridin standard 80% (v/v) methanol solution. Both showed maximum absorbance at 265 nm, which was different from those of kakkalide (270 nm) and puerarin (250 nm), indicating that tectoridin was more representative for total isoflavone of *P. lobata *flower than puerarin.

The composition of the mobile phase was optimized by comparing solvents and gradient profiles. The methanol mobile phase showed a baseline drift whereas acetonitrile mobile phase produced better separation and shorter run time. Retention time (t_R_) of tectoridin and 6"-O-xylosyl-tectoridin were 22.4 min and 18.2 min with methanol/water as mobile phase, whereas t_R _of the two isoflavone were 17.7 min and 15.6 min respectively with acetonitrile/water. Samples were eluted with a linear gradient from 15% to 50% acetonitrile/water over 35 min, then from 50% back to 15% over 36 min. The column was then washed for 4 min with this mobile phase ratio to the original conditions. Each HPLC run was within 40 minutes.

**Figure 2:**
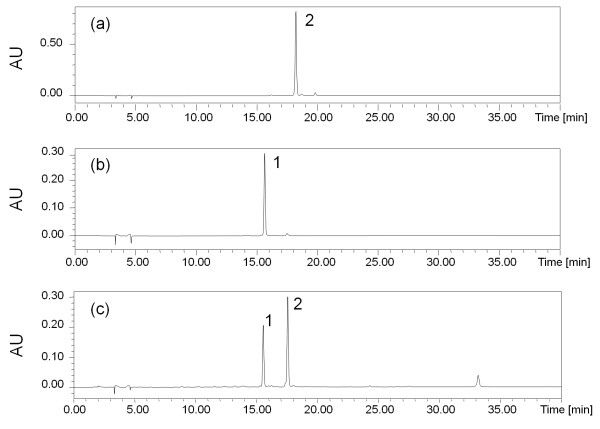
**Representative HPLC chromatograms of standards and *P. lobata *flower samples**. (a) 6"-O-xylosyl-tectoridin standard. (b) tectoridin standard. (c) a sample of *P. lobata *flower from Jiangxi province. Peaks: (1) 6"-O-xylosyl-tectoridin, (2) tectoridin.

Resolutions between tectoridin, 6"-O-xylosyl-tectoridin and other peaks were larger than 1.5 (Figure 2).

### Calibration, LOD and LOQ

The integrated chromatographic peak areas were plotted against the corresponding concentration of the standard solutions to obtain the calibration curves. The peak area response was linear for tectoridin in the 11.8-236.4 μg/mL range with a correlation coefficient of 0.9996 (P < 0.001), and for 6"-O-xylosyl-tectoridin in the 10.33-185.99 μg/mL range with a correlation coefficient of 0.9984 (P < 0.001) respectively. The regression equations for the linear portion of the standard curve of tectoridin and 6"-O-xylosyl-tectoridin were *y *= 34920*x*-1156.5 and *y *= 30731*x*-216635 respectively (*y *refers to the peak area, *x *concentration of the reference).

The LOD and LOQ for tectoridin were 24 ng/mL and 60 ng/mL, 80 and 332 ng/mL for 6"-O-xylosyl-tectoridin respectively.

The results indicated a linear correlation between the peak area and concentration. Moreover, the method was sufficiently sensitive for the determination.

### Precision

The instrument precision was assessed with intra-day and inter-day analyses using six injections of the standard solutions in duplicates. The intra-day and inter-day RSDs of the two components were less than 2% (Table 1).

**Table 1:** Precision of determination for the standards of two major isoflavones

Compounds	Concentration (μg/mL)	Intra-day RSD% (*n *= 6)	Inter-day RSD% (*n *= 6)
	30.5	1.90	1.97
Tectoridin	60.9	1.03	1.86
	118.5	0.82	1.52
	38.1	1.65	1.98
6"-O-xylosyl-tectoridin	69.4	0.78	1.95
	129.7	0.72	1.41

### Repeatability

RSDs of tectoridin and 6"-O-xylosyl-tectoridin of six independently prepared extracts of *P. lobata *flower from Henan were 0.90% and 0.78% respectively, indicating that the conditions used in the quantitative analysis were satisfactory.

### Recovery

Aliquots of the two isoflavone standards were added to *P. lobata *flower from Henan. The mixtures were then extracted and analyzed with the described HPLC method. The average recoveries were 102.7-103.7% for tectoridin and 95.7-103.2% for 6"-O-xylosyl-tectoridin (RSDs < 3%), indicating that the method was sufficiently accurate for the determination.

### Sample analysis

Means comparison of extraction yields and isoflavone determination results was performed according to one-way ANOVA. No significant difference in extraction yield was found among samples from the eleven provinces (P = 0.096), and extracts from northern China contained more isoflavones than those from southern China (P < 0.001 for tectoridin and P = 0.023 for 6"-O-xylosyl-tectoridin) whereas extracts from Guangdong had less tectoridin but more 6"-O-xylosyl-tectoridin comparing to those from northern China (Table 2).

**Table 2:** Determination of tectoridin and 6"-O-xylosyl-tectoridin in *P. lobata *flowers from eleven Chinese provinces

Province	Extraction yield (%)	Tectoridin (mg/g, *n *= 3)	6"-O-xylosyl-tectoridin (mg/g, *n *= 3)
Hebei	31.04	40.35 (0.88)	39.03 (0.77)
Shanxi	31.10	38.26 (0.90)	41.89 (0.74)
Shandong	33.78	26.46 (0.78)	30.90 (0.72)
Henan	32.02	43.28 (0.79)	38.43 (0.82)
Jiangsu	31.16	36.92 (0.83)	32.69 (0.43)
Anhui	33.32	30.50 (0.69)	30.63 (0.63)
Hubei	34.85	30.48 (0.60)	48.23 (0.95)
Jiangxi	34.24	10.00 (0.14)	18.61 (0.26)
Hunan	31.70	19.81 (0.44)	25.91 (0.31)
Guangxi	32.80	14.47 (0.26)	11.08 (0.25)
Guangdong	32.85	14.85 (0.28)	37.03 (1.05)

Extraction yield is the ratio of the amount of dry extract actually obtained, to the amount of dry herb samples used for extraction.

Data are expressed as mean (SD).

SD: standard deviation

Means comparison was performed according to one-way ANOVA with *α *= 0.05.

P > 0.05 for comparing extraction yields.

P < 0.05 for comparing two major isoflavones in samples from northern and southern provinces.

## Discussion

The HPLC method developed in this study demonstrated better separation of isoflavones and better peak shapes than those offered in previous studies [12]. Moreover, the detection wavelength described in a previous study was 262 nm [15] whereas this study found that 265 nm was the maximum absorbance wavelength for both *P. lobata *flower extract and tectoridin standard solution. Therefore, tectoridin was more representative for total isoflavones of *P. lobata *flower and 265 nm as the detection wavelength was better than 262 nm.

In Chinese medicine, medicinal herbs with high quality and efficacy from certain geographical locations are referred to as *Daodi *('genuine'). For example, *Frifllaria thuubergii *Miq. (*Zhebeimu*), a cough medicine, is only considered *Daodi *if it is from Zhejiang province, China [1]. However, no such preferential origin is currently available for *P. lobata *flowers. The present study suggests that *P. lobata *flowers from northern China may be preferred to those from southern China.

Large-scale studies with more samples will have to be carried out to confirm this variation in isoflavones of *P. lobata *flower, which is likely to have an impact on the improvement in quality and efficacy of the medicines prepared from this herb.

## Conclusion

The present study develops a new HPLC method for the determination of the two main isoflavones in *P. lobata *flowers, namely tectoridin and 6"-O-xylosyl-tectoridin. The results showed that *P. lobata *flowers from northern China contained more tectoridin and 6"-O-xylosyl-tectoridin than those from southern China.

## Competing interests

The authors declare that they have no competing interests.

## Authors' contributions

MY and YT conceived, designed and coordinated the study. MY developed the HPLC method. YL collected the materials, helped develop the HPLC method, analyzed the samples and drafted the manuscript. GQL and FCPL helped design and improved the study, drafted the manuscript and gave many important advices on research methodology. All authors read and approved the final version of the manuscript.
